# Antibacterial and Immunosuppressive Effects of a Novel Marine Brown Alga-Derived Ester in Atopic Dermatitis

**DOI:** 10.3390/md22080354

**Published:** 2024-07-30

**Authors:** Hyun Soo Kim, Jeong Won Ahn, Na Reum Ha, Kongara Damodar, Su Kil Jang, Yeong-Min Yoo, Young Soo Gyoung, Seong Soo Joo

**Affiliations:** 1 College of Life Science, Gangneung-Wonju National University, 7 Jukheon-gil, Gangneung, Gangwon 25457, Republic of Korea; gustn4609@gwnu.ac.kr (H.S.K.);; 2 Huscion MAJIC R&D Center, 331 Pangyo-ro, Seongnam, Gyeonggi 13488, Republic of Koreakongaradamu@gmail.com (K.D.);

**Keywords:** atopic dermatitis, *Hizikia fusiformis*, fatty acid ester, *Staphylococcus aureus*, immunomodulation, anti-inflammation, anti-bacterial

## Abstract

Atopic dermatitis (AD) is a chronic skin condition that is characterized by dysregulated immune responses and a heightened risk of *Staphylococcus aureus* infections, necessitating the advancement of innovative therapeutic methods. This study explored the potential of (6Z,9Z,12Z,15Z)-(2R,3R,4R,5R)-2,3,4,5,6-pentahydroxyhexyl octadeca-6,9,12,15-tetraenoate (HSN-S1), a compound derived from the marine alga *Hizikia fusiformis*, which shows anti-inflammatory, antimicrobial, and immunomodulatory properties. HSN-S1 was isolated and characterized using advanced chromatographic and spectroscopic methods. Its efficacy was evaluated via in vitro assays with keratinocytes, macrophages, and T cells to assess cytokine suppression and its immunomodulatory effects; its antibacterial activity against *S. aureus* was quantified. The in vivo effectiveness was validated using a 2,4-dinitrochlorobenzene-induced AD mouse model that focused on skin pathology and cytokine modulation. HSN-S1 significantly reduced pro-inflammatory cytokine secretion, altered T-helper cell cytokine profiles, and showed strong antibacterial activity against *S. aureus*. In vivo, HSN-S1 alleviated AD-like symptoms in mice and reduced skin inflammation, transepidermal water loss, serum immunoglobulin-E levels, and Th2/Th17 cytokine outputs. These findings suggest HSN-S1 to be a promising marine-derived candidate for AD treatment, as it offers a dual-target approach that could overcome the limitations of existing therapies, hence warranting further clinical investigation.

## 1. Introduction

Atopic dermatitis (AD), widely recognized as eczema, is a multifaceted chronic inflammatory skin disorder, characterized by intense pruritus and recurrent inflammation. The pathogenesis of AD is complex and involves the interplay between genetic, environmental, and immunological factors. The disease is characterized by a dysregulated immune response, initially dominated by T helper 2 (Th2) cells during the acute phase, which shifts towards T helper 1 (Th1) cell predominance in chronic conditions. This imbalance contributes significantly to the persistence and severity of AD [1,2,3]. The combination of these immunological challenges causes patients with AD to exhibit compromised skin barriers and reduced antimicrobial peptide levels, which predisposes them to frequent infections. Among these, colonization by *Staphylococcus aureus* is particularly detrimental, as this bacterium acts as a potent superantigen and exacerbates AD symptoms [4,5]. Therefore, therapeutic strategies have evolved to involve restoring the skin barrier and immune modulation along with targeting microbial pathogens that are integral to AD pathogenesis [6,7]. Conventional treatments include topical corticosteroids, calcineurin inhibitors, antihistamines, antibiotics, phototherapy, and systemic immunosuppressants in severe cases [8,9]. Despite their efficacy, these treatments often fail to provide sustained relief or may cause undesirable side effects, underscoring the urgent need for innovative therapeutic options [10,11,12].

Emerging evidence has indicated the therapeutic value of long-chain polyunsaturated fatty acids (LC-PUFAs) and monosaccharides. LC-PUFAs have anti-inflammatory, antibacterial, and immunomodulatory properties; in particular, they can modulate T cell responses and disrupt the cell membranes of Gram-positive bacteria, such as *S. aureus* [13,14,15]. Similarly, monosaccharides have shown promise in regulating the gut microbiota and enhancing skin health via topical applications, reflecting the intricate interconnection between the gut–skin axis and AD [16]. This study introduced (6Z,9Z,12Z,15Z)-(2R,3R,4R,5R)-2,3,4,5,6-pentahydroxyhexyl octadeca-6,9,12,15-tetraenoate (HSN-S1), a novel LC-PUFA conjugated with a sugar moiety derived from *Hizikia fusiformis*, a marine alga known for its bioactive compounds. HSN-S1 represents a groundbreaking approach to AD treatment, as it targets both microbial components and dysregulated immune responses that are characteristic of the disease. Our research utilized comprehensive in vitro and in vivo models to examine HSN-S1’s dual effect: combating bacteria and regulating immune responses, thereby presenting a potential new treatment option for managing AD. Through this exploration, we aimed to highlight the untapped potential of marine-derived compounds in addressing the multifactorial challenges of AD and to pave the way for future innovative treatments.

## 2. Results and Discussion

### 2.1. Isolation of HSN-S1 via Bioactivity-Guided Approach

The bioactivity-guided isolation of HSN-S1 from *H. fusiformis* has demonstrated its potential as an innovative therapeutic agent for AD. AD is characterized by the complex interplay between the innate and adaptive immune systems [17]. As shown in Table S3, Fraction 3 inhibited NF-κB activation by up to 79.5% in LPS-stimulated THP-1 Lucia™ NF-κB luciferase cells, and up to 69.5% in Anti-CD3ε-stimulated splenocytes. This underscores the potential of HSN-S1 to mitigate the central inflammatory milieu of AD. Therefore, through bioactivity-guided fractionation, the HSN-S1 compound was successfully isolated. The broad-spectrum inhibitory effect of HSN-S1 across various cellular models, including mouse splenocytes and THP-1 NF-κB cells, reinforces its applicability as a versatile anti-inflammatory agent that can target diverse immunological facets implicated in AD. This study provides a coherent narrative on the mode of action of HSN-S1, highlighting its potential to restore balance during immune dysregulation at the core of AD pathogenesis while offering robust foundations for its therapeutic application in immune-mediated dermatological conditions. Further elucidating the potential of HSN-S1 to alleviate inflammation and restore immune homeostasis provides a promising avenue for future AD research and treatment, warranting further clinical investigation into its efficacy and application as a novel therapeutic agent.

### 2.2. Structural Characterization of HSN-S1

HSN-S1 was identified as a pale yellowish oil. The molecular formula was determined to be C_24_H_40_O_7_ by HRESIMS at *m*/*z* 463.2673 (calculated 463.2666 for C_24_H_40_NaO_7_ [M + Na^+^]), corresponding to five double-bond equivalents. The infrared spectrum revealed the existence of three key functional groups: hydroxyl (3381 cm^−1^), carbonyl (1741 cm^−1^), and -C=C- (1635 cm^−1^). Detailed 1D nuclear magnetic resonance (NMR) (^1^H, ^13^C, and distortionless enhancement by polarization transfer) analysis of HSN-S1 revealed the presence of one sp^3^ methyl, ten sp^3^ methylene, four sp^3^ methine, eight sp^2^ methine, and one ester carbonyl carbon. In the ^1^H NMR spectrum, eight protons (*δ*_H_ 5.37–5.35 (1H, m), 5.35–5.34 (1H, m), 5.34–5.30 (5H, m), and 5.30–5.28 (1H, m)), and their corresponding carbon signals (*δ*_C_ 131.5, 129.6, 128.1, 128.0, 127.8, 127.7, 127.7, 126.9) suggested the presence of four *cis*-olefinic double bonds (Table 1). A characteristic clear triplet at *δ*_H_ 0.92 (3H, t, *J* = 7.7 Hz) was designated the -CH_3_ terminal group of an unsaturated ω-3 fatty acid, which typically appeared in a narrow range of 0.87–0.88 ppm in ω-6 and ω-9 unsaturated fatty acids. Signals at *δ*_H_ 2.82–2.79 (6H, m) and corresponding signals at *δ*_C_ 25.2, 25.2, 25.1 disclosed the presence of three bis-allylic methylene groups. The ^13^C NMR chemical shifts between δ_C_ 63.8 and 71.2 signified the presence of a sugar alcohol moiety in HSN-S1, which was typical for oxygen-attached carbons. The signals at δ_H_ 4.28 (1H, dd, *J* = 11.2, 2.8 Hz), 3.94 (1H, dd, *J* = 11.2, 7.0 Hz), 3.39 (1H, dd, *J* = 11.2, 6.3 Hz), and 3.61 (1H, dd, *J* = 11.2, 3.5 Hz), along with corresponding δ_C_ values of 66.9 and 63.8, provided evidence for the presence of two hexitol methylene groups. Hexitol exhibited four methine groups, detected at *δ*_H_ 3.66 (1H, m), 3.55 (2H, d, *J* = 9.1 Hz), 3.45 (1H, m) with corresponding δ_C_ values of 68.3, 69.5, 71.2, and 71.2 in the ^1^H and ^13^C NMR spectra, respectively.

**Figure 1:**
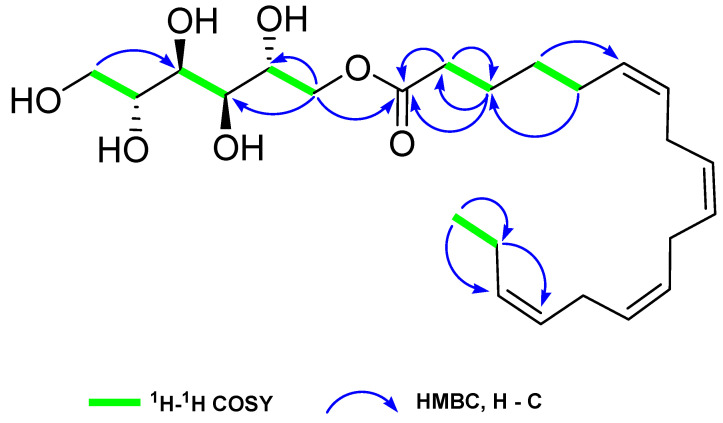
Key ^1^H-^1^H COSY (green bold lines) and HMBC (blue arrows) correlations of HSN-S1.

This composition was further corroborated using correlation spectroscopy (COSY) and heteronuclear multiple bond correlation (HMBC) analyses, which mapped the HSN-S1 fatty acids and hexitol components via intricate cross-peaks and correlations (Figure 1). The COSY analysis delineated two primary spin systems: one elucidating the fatty acid chain from C-2 (*δ*_C_ 33.4) to C-18 (*δ*_C_ 14.1) by a series of correlations from H-2 (*δ*_H_ 2.30) to H-18 (*δ*_H_ 0.92), and another outlining the hexitol structure from C-1’ (*δ*_C_ 66.9) to C-6’ (*δ*_C_ 63.8) by the correlations from H-1’ (*δ*_H_ 4.28 and 3.94) to H-6’ (*δ*_H_ 3.39 and 3.61) through H-2’ (*δ*_H_ 3.66), H-3’ (*δ*_H_ 3.55), H-4’ (*δ*_H_ 3.55), and H-5’ (*δ*_H_ 3.45). In HMBC, correlations such as H-18/C-17, C-16; H-17/C-18, C-16, C-15 reconfirmed the ω-3 fatty acid functionality. Correlations like H-14/C-15, C-13; H-11/C-12, C-10; H-8/C-7, C-9 indicated three bis-allylic moieties. In the hexitol moiety, Ha-1’/C-1, C-2’; Hb-1’/C-1, C-2’ HMBC correlations were significant. The information on the structural elucidation is organized in the Supplementary Materials.

To verify the stereochemistry of the hexitol component, base-mediated hydrolysis was performed, which isolated mannitol, as evidenced by comparative NMR (Figures S11 and S12). The unique composition of HSN-S1 was characterized by the integration of mannitol and a ω-3 fatty acid; this lays a solid foundation for further exploration of its bioactivity, particularly in AD.

### 2.3. Effectiveness of HSN-S1 on Keratinocytes

The assessment of HSN-S1’s anti-AD activities on keratinocytes revealed interesting therapeutic implications for controlling this chronic skin disorder. Keratinocytes, the predominant cell type within the human epidermis, play an essential role in maintaining skin barrier integrity and body protection from various external threats, including environmental stimuli, microbes, and pathogens [18,19]. These cells are critically involved in the immune response and contribute significantly to the inflammatory processes involved in AD. TNF-α and interferon-γ (IFN-γ) orchestrate a series of cellular changes that closely resemble the inflammatory state observed in AD, and are central to the inflammatory response in keratinocytes [17,19]. The interaction between TNF-α and IFN-γ is particularly detrimental, and increases chemoattractant production for monocytes and T cells, upregulates adhesion molecules, and reduces the endothelial barrier integrity. These alterations collectively increase skin permeability, a characteristic feature of AD, which further exacerbates the condition [20]. The generation of pro-inflammatory cytokines and chemokines, viz., tumor necrosis factor-α (TNF-α), monocyte chemoattractant protein-1 (MCP-1), and C-X-C motif chemokine ligand 10 (CXCL10), by keratinocytes is a pivotal factor in the perpetuation of AD [18,19,20,21]. These mediators aid in recruiting inflammatory cells to the skin, and contribute to the breakdown of the skin barrier; thus, targeting these molecules is vital in AD treatment.

**Figure 2:**
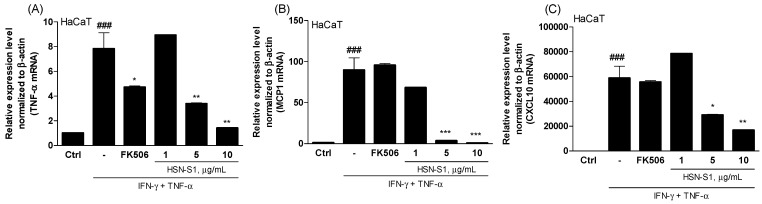
HSN-S1 reduces proinflammatory gene expression in IFN-γ/TNF-α-stimulated HaCaT cells. Cells were pretreated with HSN-S1 for 1 h, then exposed to IFN-γ (5 ng/mL) and TNF-α (15 ng/mL) to mimic an AD environment. The anti-inflammatory effects of HSN-S1 were evaluated by measuring the expression of (**A**) TNF-α, (**B**) MCP-1, and (**C**) CXCL10 20 h post-stimulation. Data are shown as mean ± SEM from four independent experiments. Substantial reductions in gene expression were observed with HSN-S1 (### *p* < 0.001 vs. control; * *p* < 0.05, ** *p* < 0.01, *** *p* < 0.001 vs. IFN-γ/TNF-α group). FK506 (1 ng/mL) served as a standard anti-inflammatory compound for comparison.

Before analyzing the efficacy of HSN-S1, we assessed the half-maximal cytotoxicity concentration (CC_50_) of AD-related cells (HaCaT, RAW264.7, splenocytes, naive CD4^+^ Th, Jurkat, RBL-2H3, Jurkat-Lucia™ NFAT) and conducted the studies under non-toxic conditions (Table S1). HSN-S1 treatment applied to HaCaT cells, a keratinocyte model stimulated with IFN-γ and TNF-α, significantly suppressed key pro-inflammatory cytokines and chemokines. The results demonstrated a *p*-value < 0.05, indicating a statistically significant difference between the HSN-S1 treated group and control groups, thereby underscoring the efficacy of HSN-S1 in modulating inflammatory responses. (Figure 2). This finding suggests that HSN-S1 can mitigate the inflammatory responses initiated by keratinocytes in AD. HSN-S1 inhibits the secretion of these critical inflammatory mediators, directly addressing a fundamental mechanism that drives AD pathogenesis. Consequently, the anti-inflammatory effects of HSN-S1 in keratinocytes display its potential as an effective therapeutic agent in AD. Therefore, this study elucidates the role of HSN-S1 in modulating keratinocyte-mediated inflammatory pathways and supports the broader application of marine-derived compounds in treating dermatological conditions labeled by inflammation and impaired skin barrier function.

### 2.4. Effect of HSN-S1 on Macrophage-Mediated Inflammation

**Figure 3:**
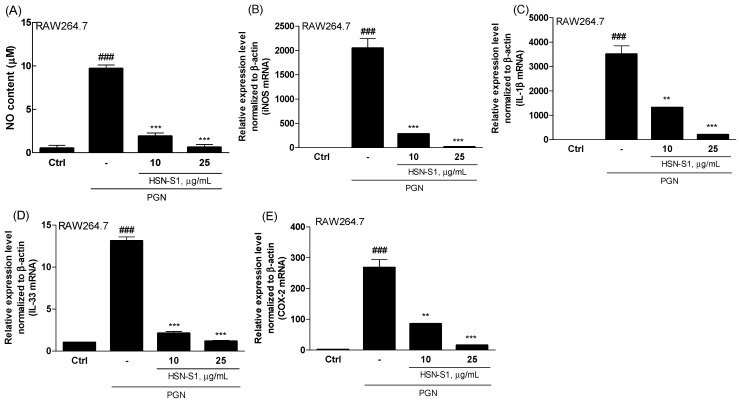
HSN-S1 mitigates inflammatory responses in PGN-stimulated RAW264.7 cells. Cells were pretreated with HSN-S1 for 1 h, then exposed to PGN 10 μg/mL to mimic an inflammation environment. The capacity of HSN-S1 to suppress the expression of critical inflammatory factor (NO) and genes (iNOS, IL-1β, IL-33, COX-2) in RAW264.7 macrophage cells was evaluated. Gene expression was quantified 20 h post-PGN exposure, with results indicating significant downregulation in the presence of HSN-S1. Data are shown as mean ± SEM from four independent experiments. Substantial reductions in gene expression were observed with HSN-S1 (### *p* < 0.001 vs. control; ** *p* < 0.01, *** *p* < 0.001 vs. PGN group).

**Figure 4:**
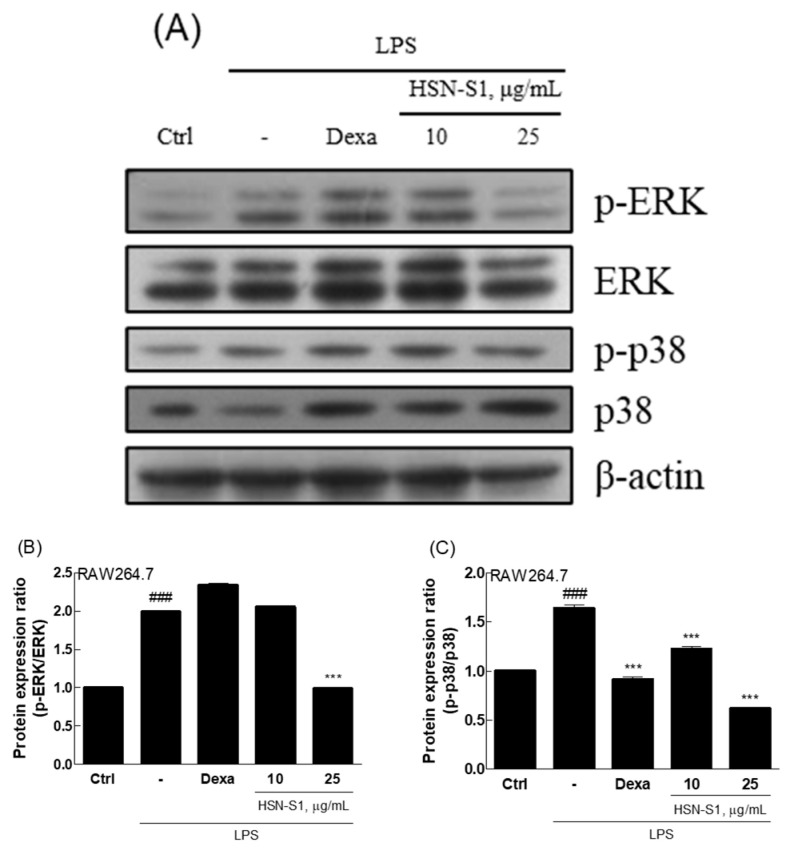
HSN-S1 inhibits MAPK pathway activation in LPS-challenged RAW264.7 cells. Cells were pretreated with HSN-S1 for 1 h, then exposed to LPS 1 μg/mL to mimic an inflammation environment. Protein expression was quantified 1 h post-LPS exposure. Panel (**A**) displays the Western blot results. Panels (**B**,**C**) quantify the ratio of phosphorylated to total ERK (p-ERK/ERK) and p38 (p-p38/p38), respectively. Results, represented as mean ± SEM from three independent experiments, demonstrated a significant reduction in MAPK pathway activation by HSN-S1 (### *p* < 0.001 vs. control; *** *p* < 0.001 vs. LPS group). Dexamethasone (Dexa; 1 μM) served as a standard anti-inflammatory compound for comparison.

**Figure 5:**
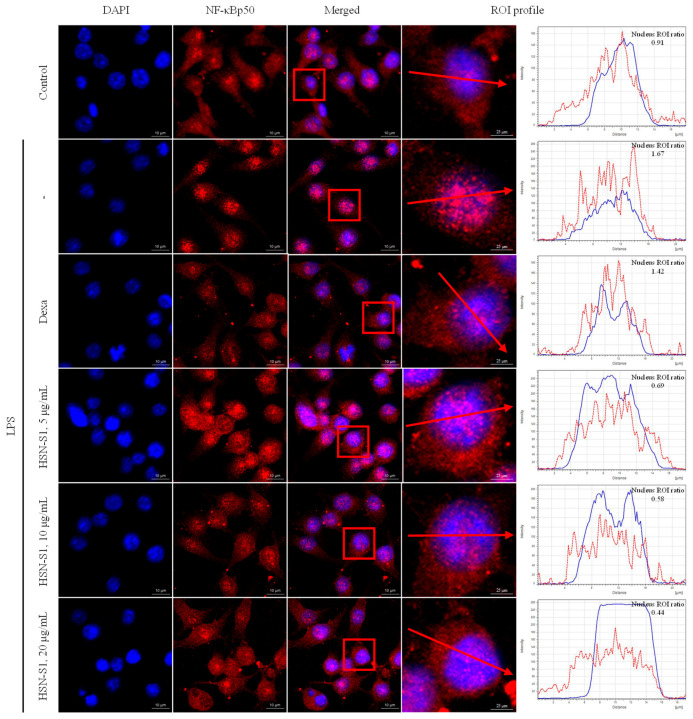
HSN-S1 blocks NF-κB nuclear translocation in LPS-stimulated RAW264.7 cells. Cells were first treated with HSN-S1 before 1 μg/mL of LPS induction to assess HSN-S1 efficacy in preventing NF-κB translocation from the cytoplasm to the nucleus. Dexamethasone (Dexa; 1 μM), was used as a reference anti-inflammatory agent for comparative analysis. The figure employs region of interest (ROI) analysis, depicted within a red box and indicated by a red arrow, to precisely evaluate the inhibition of NF-κB translocation by HSN-S1.

The anti-AD effects of HSN-S1 extend to macrophages, a field previously overshadowed by the regulatory roles of T cells in AD research. Macrophages are pivotal in chronic inflammation; hence, our study sheds light on their potential role in AD, particularly considering their capacity to produce inflammatory mediators [22]. Utilizing mouse macrophage RAW264.7 cells, we investigated the response to peptidoglycan (PGN), a component of Gram-positive bacteria known to activate inflammatory pathways via toll-like receptor 2 [23]. PGN stimulation causes the secretion of various pro-inflammatory cytokines and substances, including nitric oxide (NO), IL-1β, IL-33, and cyclooxygenase-2 (COX-2), which are instrumental in initiating and amplifying inflammatory responses across several cell types [23,24,25]. Our findings revealed that HSN-S1 significantly reduced PGN-induced NO production in RAW264.7 cells, highlighting its potential to mitigate macrophage-mediated inflammation (Figure 3A). Furthermore, the ability of HSN-S1 to suppress the expression of genes encoding pro-inflammatory cytokines (Figure 3B,C) and inflammatory mediated enzyme (Figure 3D,E) underscores its efficacy in dampening the inflammatory cascade. This dual action suggests a broader immunomodulatory effect of HSN-S1, extending beyond inflammation suppression to potentially prevent immune cell recruitment to the skin, thereby offering a strategy to counteract chronic inflammatory responses in AD. The mitogen-activated protein kinase (MAPK) pathway, essential for transmitting extracellular signals to the cell interior, plays a significant role in inflammation and has been identified as an HSN-S1 target. The inhibition of the classical MAPK pathways, including extracellular signal regulated kinase (ERK), jun amino-terminal kinase/stress-activated protein kinase (JNK/SAPK), and p38 kinase, by HSN-S1 (Figure 4) further elucidated the mechanism of suppression of AD-related genes and inflammatory responses. In relation to NF-κB signaling, HSN-S1 demonstrated superior ability to prevent the nuclear translocation of the NF-κB p50 subunit in LPS-stimulated RAW264.7 cells, which are stimulated through the TLR4 receptor, a well-known pathway in macrophage inflammatory response [26]. This effect was notable when compared to the anti-inflammatory corticosteroid dexamethasone (Figure 5).

These findings suggest that HSN-S1 is a compelling anti-inflammatory agent with a distinct capacity to modulate macrophage-driven responses in AD, and it inhibits key inflammatory pathways and cytokine production, providing a multifaceted approach for addressing the complex immunological environment of AD.

### 2.5. Immunomodulatory Actions of HSN-S1 in Th Cells

**Figure 6:**
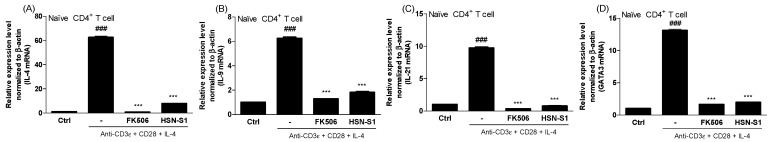
HSN-S1 dampens inflammatory signaling in T cells. Cells were pre-treated with HSN-S1 (25 μg/mL) before co-stimulation with IL-4 (50 ng/mL) and the T cell activation antibodies anti-CD3ε and anti-CD28 (0.5 μg/mL each). We focused on the expression of cytokines (IL-4, IL-9, and IL-21, (**A**–**C**)) and transcription factor GATA3 (**D**), which are crucial for T cell differentiation and function, 20 h after stimulation. Results, represented as mean ± SEM from four independent studies, revealed that HSN-S1 significantly inhibited inflammatory gene expression (### *p* < 0.001 vs. control; *** *p* < 0.001 vs. stimulated group without HSN-S1). FK506 (1 ng/mL) was used as a reference anti-inflammatory agent.

**Figure 7:**
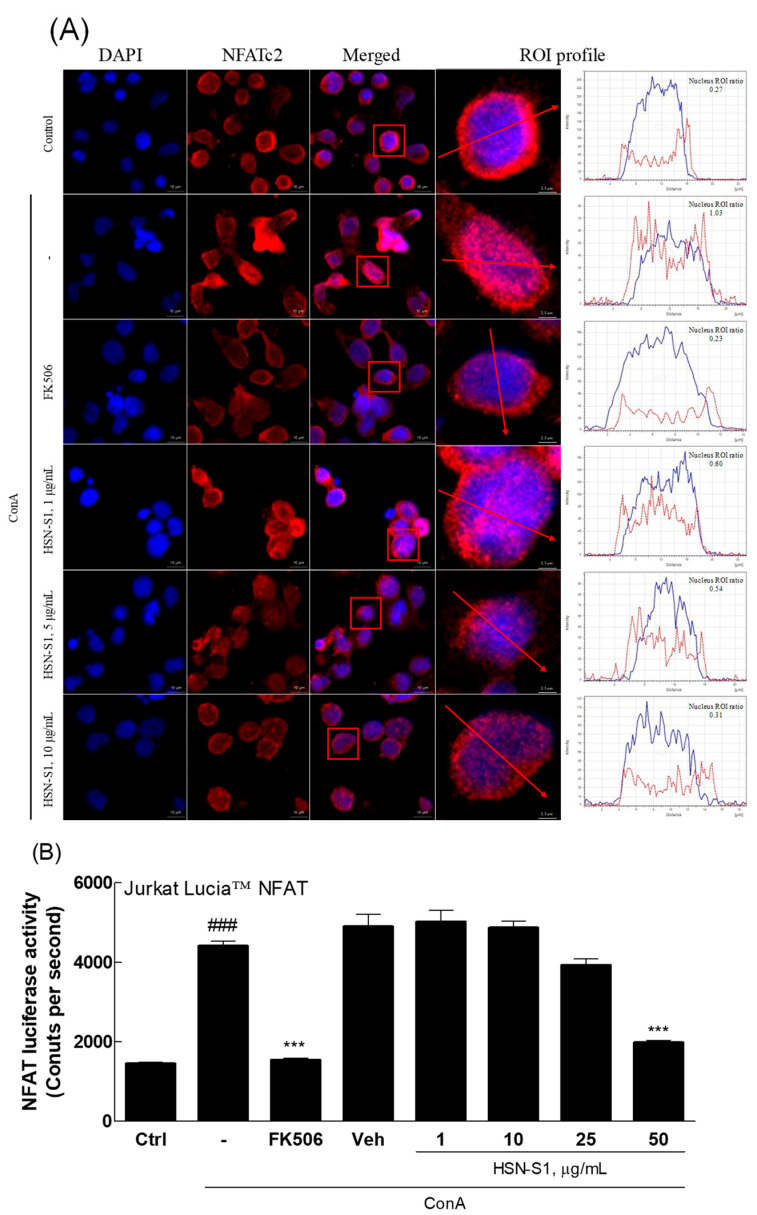
HSN-S1 prevents NFATc2 nuclear translocation in activated Jurkat T cells. The capability of HSN-S1 to inhibit NFATc2 nuclear translocation, a pivotal event in T-cell activation, using Jurkat cells as a model system is shown. Cells were pre-treated with HSN-S1, followed by 10 μg/mL of ConA stimulation, a condition that activates the NFATc2 signaling pathway. (**A**) One hour after the ConA administration, NFATc2 nuclear migration was analyzed. The analysis, focused within a red-boxed region of interest (ROI) marked by a red arrow, quantitatively evaluated NFATc2 localization. (**B**) The effect of HSN-S1 on NFAT pathway engagement was examined using Jurkat Lucia™ NFAT cells that express luciferase in response to NFAT activation. The Jurkat Lucia™ NFAT cells were treated with the sample, followed by the administration of 10 μg/mL ConA. The analysis was conducted after a 20 h response period. Results, depicted as mean ± SEM from six independent experiments, revealed significant suppression of NFAT-driven luciferase expression by HSN-S1 (### *p* < 0.001 vs. control; *** *p* < 0.001 vs. ConA group). FK506 (1 ng/mL) was used as a reference anti-inflammatory agent.

The anti-AD effects of HSN-S1 in Th cells impart significant insights into its potential for AD. CD4^+^ T cells play a key role in AD immunopathology, interacting with various immune cells to activate and differentiate into Th2 and Th17 subsets, which drive the inflammatory response [27,28]. HSN-S1 displayed its impact by suppressing Th2-mediated cytokines (IL-4, IL-9, and IL-21) and transcription factors (GATA3), crucial for Th2 and Th17 cell differentiation (Figure 6). This effect mirrors the action of FK506, a known anti-AD medication, demonstrating HSN-S1’s efficacy in modulating the Th cell-mediated immune response in AD. A key finding of this study is HSN-S1’s modulation of the NFAT pathway, which is critical for T cell activation and differentiation. The pathway is initiated through T cell receptor engagement, calcium influx, and calcineurin activation [29]. The ability of HSN-S1 to prevent the nuclear translocation of NFAT in concanavalin A (ConA)-stimulated Jurkat cells (Figure 7A) and inhibit NFAT luciferase activity (Figure 7B) validates its suppressive effect on T cell activation while highlighting its potential in regulating the excessive immune response, which is characteristic of AD.

These findings underscore HSN-S1’s potential as a novel therapeutic agent that can target multiple aspects of the immune response implicated in AD. HSN-S1 addresses the complex immune dysregulation that is central to AD pathogenesis via modulating Th cell activity and inhibiting critical signaling pathways such as NFAT. The broad-spectrum immunomodulatory effects of HSN-S1 that encompass Th1 and Th2 response suppression, and key cytokine and transcription factor regulation, warrant further investigation into its mechanisms of action and potential clinical applications for AD treatment.

### 2.6. HSN-S1 and Mast Cell Degranulation: A β-Hexosaminidase Release Assay

**Figure 8:**
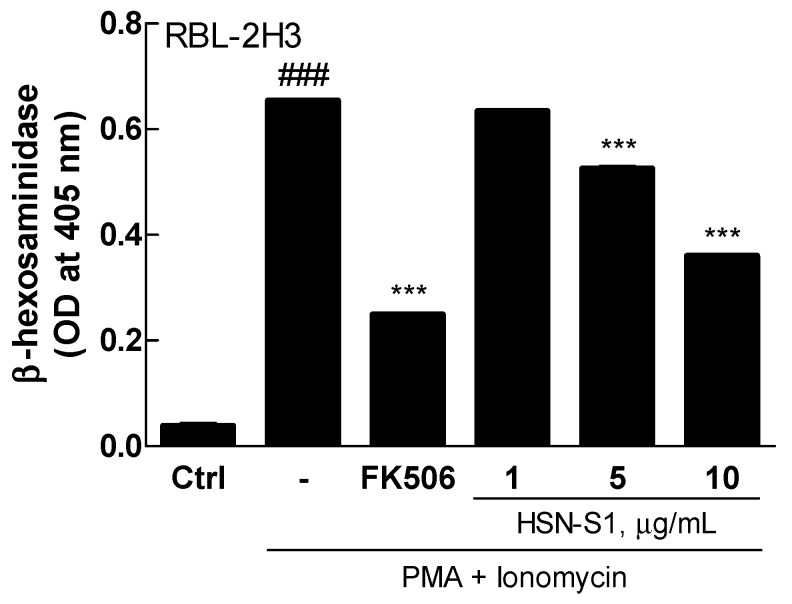
HSN-S1 reduces allergic response markers in stimulated RBL-2H3 mast cells. The anti-allergic efficacy of HSN-S1 was quantitatively assessed 3 h post-stimulation by measuring β-hexosaminidase release. Results are represented as mean ± SEM from two independent experiments, indicating a significant attenuation of β-hexosaminidase release with HSN-S1 treatment (### *p* < 0.001 vs. control; *** *p* < 0.001 vs. PMA/ionomycin group). FK506 (1 ng/mL) was used as a reference anti-inflammatory agent.

The β-hexosaminidase release assay is a crucial indicator of the anti-allergic potential of HSN-S1, particularly its ability to modulate the activity of basophils and mast cells, which are central to the pathogenesis of allergic and inflammatory diseases. These cells are abundant throughout the body and play key roles in the immune response to allergens. On activation, they release various inflammatory mediators, including histamine, β-hexosaminidase, and cytokines, which collectively contribute to the development and exacerbation of inflammatory responses [30]. Thus, the significance of the effects of HSN-S1 on these cells cannot be overstated. The concentration-dependent suppression of β-hexosaminidase release from RBL-2H3 cells, a model for rat basophilic leukemia that mimics mast cell behavior in allergic reactions, provides compelling evidence of its anti-inflammatory and anti-allergic properties. HSN-S1 effectively reduced the release of mediators that would otherwise amplify the inflammation and allergic responses via curtailing degranulation (Figure 8). The ability of HSN-S1 to suppress basophil and mast cell activity, hence mitigating the allergic inflammatory process, underscores its therapeutic potential for treating AD. AD is characterized by chronic inflammation and hypersensitivity reactions in the skin; a significant immune component drives its pathogenesis. The β-hexosaminidase release assay results suggest that HSN-S1 could offer a novel approach to AD treatment by targeting the overactive response of the immune system to external allergens.

### 2.7. Antimicrobial Activity of HSN-S1 Against S. aureus

**Figure 9:**
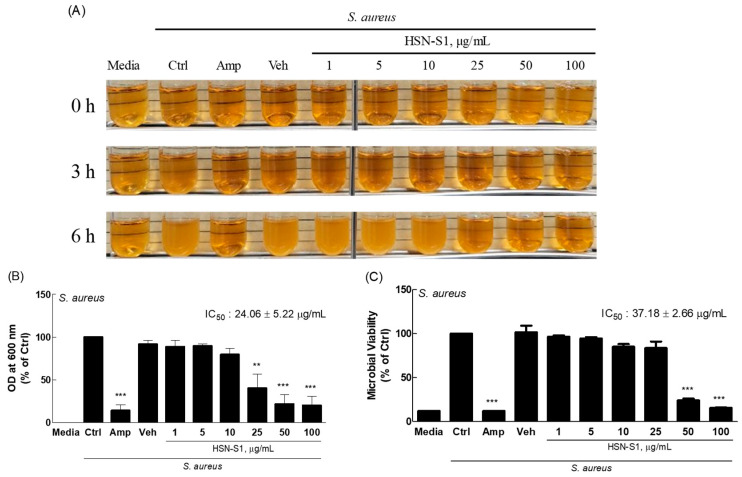
HSN-S1 inhibits the growth of *Staphylococcus aureus*. (**A**) This figure shows the changes in microbial density over time after treatment with HSN-S1 following microbial inoculation. (**B**) The effect of HSN-S1 on bacterial growth was monitored over 6 h, with graphs representing changes in bacterial density. (**C**) Comparative analysis of microbial viability revealed the effectiveness of HSN-S1 in curtailing *S. aureus* proliferation relative to the vehicle (veh) control (1% dimethyl sulfoxide) and ampicillin (Amp; 100 μg/mL), which was a benchmark for antibacterial efficacy. The results, expressed as mean ± SEM from two independent studies, indicated a significant reduction in *S. aureus* growth in the presence of HSN-S1 (** *p* < 0.01, *** *p* < 0.001 vs. control).

*S. aureus* is prevalent on the skin of patients with AD, where it exacerbates disease severity through several mechanisms. *S. aureus* initiates a cascade of inflammatory responses by adhering to keratinocytes via protein clumping factor B (ClfB) and forming colonies, and it secretes several pro-inflammatory proteins and superantigens such as protein A, lipoproteins, toxic shock syndrome toxin-1, various enterotoxin serotypes (SEA, SEB, SEC, SED, SEE, and SEG), and α and δ toxins. These factors contribute to inflammation, itchiness, and the overall aggravation of AD [31,32,33]. The skin affected by AD is characterized by dryness and occasional wounds, which becomes a fertile ground for *S. aureus* to penetrate deeper into the skin layers, triggering an immune response that worsens the condition. The discovery that HSN-S1 could effectively inhibit *S. aureus* growth (Figure 9), with a half-maximal inhibitory concentration of 24.06 ± 5.22 μg/mL, marks a significant step towards a more integrated approach to managing AD. Traditional treatments often rely on antibiotics such as gentamicin, fusidic acid, and mupirocin to control *S. aureus* infections, which, while effective, have limitations, such as the potential for antibiotic resistance and side effects [34]. We investigated the antibacterial activity of HSN-S1 against *S. aureus*, a bacterium that exacerbates AD through inflammatory responses. Although *S. epidermidis* also plays a role in skin health, our study focused on *S. aureus* due to its significant impact on AD symptoms. Future research will explore HSN-S1’s effect on *S. epidermidis*.

HSN-S1 has dual functionality—both combating immune dysregulation inherent in AD and directly inhibiting the growth of a key exacerbating bacterial agent—which positions it as a promising candidate for a novel AD treatment. Its effectiveness in suppressing *S. aureus* growth, in addition to its previously demonstrated anti-inflammatory properties, underscores its potential to serve as an alternative or adjunct to existing antibiotic regimens. This could enhance treatment outcomes for patients with AD and contribute to reducing the reliance on traditional antibiotics, mitigating the risks associated with antibiotic resistance. Additional studies and clinical evaluations are necessary to verify HSN-S1’s efficacy and safety as a treatment option for AD.

### 2.8. HSN-S1’s Role in Reducing Epidermal Lichenification

**Figure 10:**
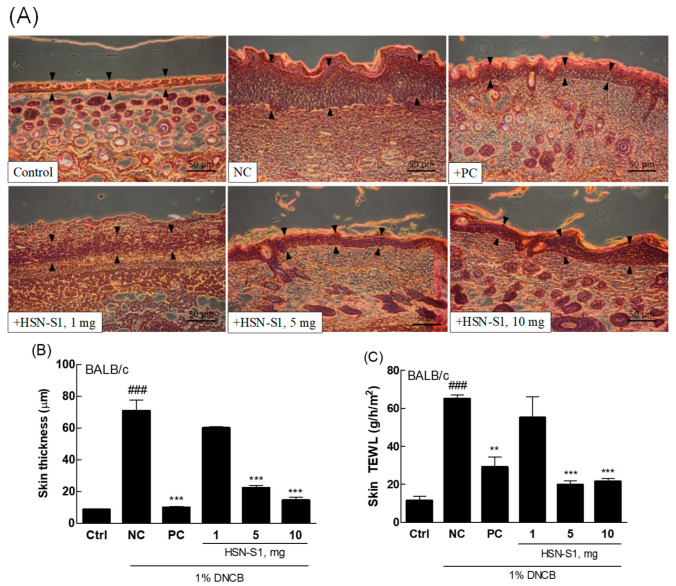
HSN-S1 ameliorated AD-like lesions in DNCB-treated BALB/c mice. (**A**) Skin tissue sections stained with hematoxylin and eosin. (**B**) Measurement of epidermal thickness in the areas indicated by black arrows. (**C**) Analysis of TEWL. Data, represented as mean ± SEM, reflect significant enhancements in skin condition and barrier performance with HSN-S1 treatment (### *p* < 0.001 vs. untreated control; ** *p* < 0.01, *** *p* < 0.001 vs. the DNCB-treated negative control group).

Evaluating the effect of HSN-S1 on cutaneous epidermal lichenification is a significant step toward understanding its therapeutic potential in AD. Lichenification is characterized by skin thickening, hyperpigmentation, and increased skin creases, and is a hallmark of chronic AD. This condition arises from a complex interplay of factors, including altered skin lipid composition, compromised moisture retention, ceramide deficiencies, impaired filaggrin function, and disturbances in skin pH levels. These alterations predispose the skin to lichenification while facilitating *S. aureus* colonization, which exacerbates the condition [35]. HSN-S1 can modulate keratin production and significantly reduce transepidermal water loss (TEWL), which offers a dual approach for mitigating the pathological processes underlying lichenification (Figure 10). HSN-S1 directly addresses the barrier function of the skin to alleviate the primary symptoms of lichenification, hence restoring epidermal barrier integrity. This is crucial for limiting the susceptibility of lichenified skin to invasion and colonization by *S. aureus*, a common aggravator of AD pathology.

Furthermore, HSN-S1 demonstrated antimicrobial activity against *S. aureus*, complementing its barrier-restoring effects, and it inhibits *S. aureus* growth, tackling a key exacerbating factor of AD, and breaks the cycle of infection and inflammation that perpetuates the disease severity. This dual action enhances skin barrier function, exerts antimicrobial effects, and positions HSN-S1 as a comprehensive therapeutic agent capable of addressing both the symptoms and contributing factors of AD. The implications of these findings are substantial, suggesting that HSN-S1 could offer a novel therapeutic option for patients with AD, particularly those with chronic lichenification and recurrent *S. aureus* infections, and it targets the underlying mechanisms of the disease on multiple fronts, promising a more holistic approach to AD management, potentially improving patient outcomes and well-being.

### 2.9. In Vivo Efficacy of HSN-S1: Th Cell-Mediated Cytokine Modulation and Serum Immunoglobulin (IgE) Suppression by HSN-S1 in AD Models

**Figure 11:**
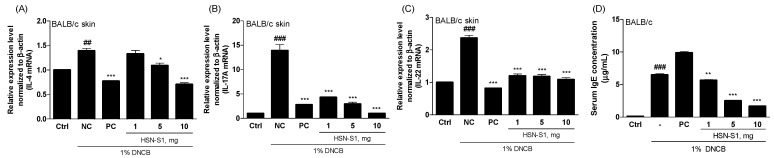
HSN-S1 reduces cytokine production by Th cells and serum IgE levels in BALB/c mice. In a DNCB-induced AD model in BALB/c mice, HSN-S1 treatment significantly altered the levels of pivotal Th cell cytokines, including IL-4 (**A**), IL-17A (**B**), and IL-22 (**C**), which are instrumental in driving inflammatory processes characteristic of AD. (**D**) The ability of HSN-S1 to reduce serum IgE levels in BALB/c mice applied with a chemically induced AD model using DNCB. Data, represented as mean ± SEM from four independent studies, demonstrate marked reductions in cytokine levels following HSN-S1 administration (## *p* < 0.01, ### *p* < 0.001 vs. untreated control; * *p* < 0.05, ** *p* < 0.01, *** *p* < 0.001 vs. DNCB-treated negative control group).

A study of the effects of HSN-S1 on Th-mediated genes in DNCB-treated mice revealed its significant therapeutic potential in mitigating AD symptoms. Th17 cells are integral to the immune response and produce cytokines, such as IL-17A and IL-22. IL-17A is critical for macrophage activation and the subsequent production of pro-inflammatory cytokines such as TNF-α and IL-1β, which play pivotal roles in inflammatory processes. Moreover, IL-17A promotes the epithelial cells to produce IL-8 and granulocyte-macrophage colony-stimulating factor (GM-CSF), facilitating neutrophil migration and activation. Conversely, IL-22 is implicated in chronic pruritus and disrupting the skin barrier function, which exacerbates sensitization to allergens and contributes to the cycle of inflammation and itching symptoms of AD [36,37]. Although the DNCB-stimulated mouse model closely mimics the inflammatory environment of human AD and provides beneficial insights into the therapeutic potential of HSN-S1, acknowledging the limitations inherent in extrapolating these findings directly to humans is necessary. Differences in skin physiology, immune response, and disease manifestation between mice and humans may influence the efficacy and safety profiles of HSN-S1. Specifically, HSN-S1 treatment suppressed cytokines, which are typically elevated during the Th2 cell sensitization phase of AD (Figure 11A–C). It significantly reduced levels of IL-17A and IL-22, cytokines directly involved in itching and skin barrier impairment. The implications of these findings are twofold. First, HSN-S1 attenuates IL-17A and IL-22 expression, addressing two critical pathways that contribute to AD pathophysiology: inflammation and skin barrier disruption. This action alleviates the itchiness caused by allergen-induced sensitization and plays a role in restoring skin barrier integrity, which reduces the overall severity of AD symptoms. Second, HSN-S1 can modulate Th-mediated gene expression, which underscores its potential as a comprehensive therapeutic agent for targeting the complex immune dysregulation underlying AD. Considering the chronic nature of AD and the limitations of current treatments, particularly in managing severe or refractory cases, these results of HSN-S1 treatment on Th-mediated genes offer hope for a more effective and targeted approach to AD therapy.

The inhibitory effect of HSN-S1 on IgE production marks a significant advancement in AD management, a condition characterized by intense itching, which may cause severe skin damage. IgE plays a pivotal role in allergic reactions and acts as a key mediator contributing to the itching and scratching associated with AD. Elevated levels of serum IgE are closely linked to AD symptom severity, as they cause a heightened immune response, particularly in skin already compromised by lesions or infections, such as those caused by *S. aureus* [38,39]. The observed reduction in serum IgE levels after HSN-S1 treatment represents a critical therapeutic action (Figure 11D). HSN-S1 diminishes the IgE concentration, hence directly addressing a primary driver of the allergic response in AD; this reduces the urge to scratch and consequently minimizes the risk of further skin damage. This reduction in IgE levels and the associated decrease in itching aid in alleviating the immediate discomfort experienced by patients with AD while contributing to the long-term healing and restoration of the skin barrier function.

The capacity of HSN-S1 to suppress serum IgE production and its implications for AD treatment are significant. The combined antimicrobial and anti-inflammatory effects of HSN-S1 demonstrated in vitro highlight its potential as a multifaceted therapeutic agent capable of comprehensively alleviating AD symptoms. Further exploration of the mechanisms by which HSN-S1 influences IgE levels and conducting clinical trials to assess its efficacy and safety in human subjects are essential to fully leverage its potential in AD treatment.

## 3. Materials and Methods

### 3.1. Compound Extraction and Characterization

#### 3.1.1. Anti-AD Bioactivity-Guided Isolation of *H. fusiformis* Compounds

*H. fusiformis* was obtained from the east coast of South Korea in August 2019 and thoroughly cleaned, dried, and ground. The process from its extraction to the fraction where anti-AD activity was confirmed is illustrated schematically in Figure S1. The dried plant material was extracted three times with 65% ethanol at a 1:10 plant-to-solvent ratio for 6 h at 25 °C. The combined ethanol extracts were designated as HSN-S1, filtered using filter paper No.20 (Hyundai micro Co. Ltd., Seoul, Republic of Korea), and concentrated under reduced pressure. The concentrated extract was subjected to a liquid–liquid extraction process to isolate compounds with potential anti-AD activity. This process utilized a series of solvents with increasing polarity: n-hexane, ethyl acetate, and water. The fractions were evaporated to dryness. The ethyl acetate fraction, which exhibited anti-AD activity, was analyzed using high-performance liquid chromatography (HPLC, Thermo Fisher Scientific, Waltham, MA, USA). Chromatographic separation was achieved using a YMC-Triart C18 column (250 × 4.6 mm, 12 nm, S-5 μm) from YMC (Kyoto, Japan), with a Security Guard™ column (Phenomenex, Torrance, CA, USA) to protect the analytical column. To isolate the active components, the ethyl acetate fraction was subjected to preparative HPLC (prep-HPLC) using an LC-Forte/R system (YMC) equipped with a YMC Triart C18 column (250 × 20 mm, 12 nm, S-5 μm). The same elution parameters as those established during analytical HPLC were employed for isolation (the gradient conditions are shown in Table S1). The fractions obtained were concentrated and then stored at 4 °C until they were ready for further analysis. Isolated compounds were subjected to activity analysis on the separated fractions using THP-1 NF-κB and mouse splenocyte cells; they were purified using fractions that exhibited strong anti-AD efficacy in both cell types (the activity analysis data and the prep HPLC separation chromatogram are shown in Figure S2 and Table S2, respectively). HPLC and prep-HPLC analyses were conducted by measuring absorbance at 203 nm for identification.

#### 3.1.2. Chromatographic Analysis and Sample Preparation

**Figure 12:**
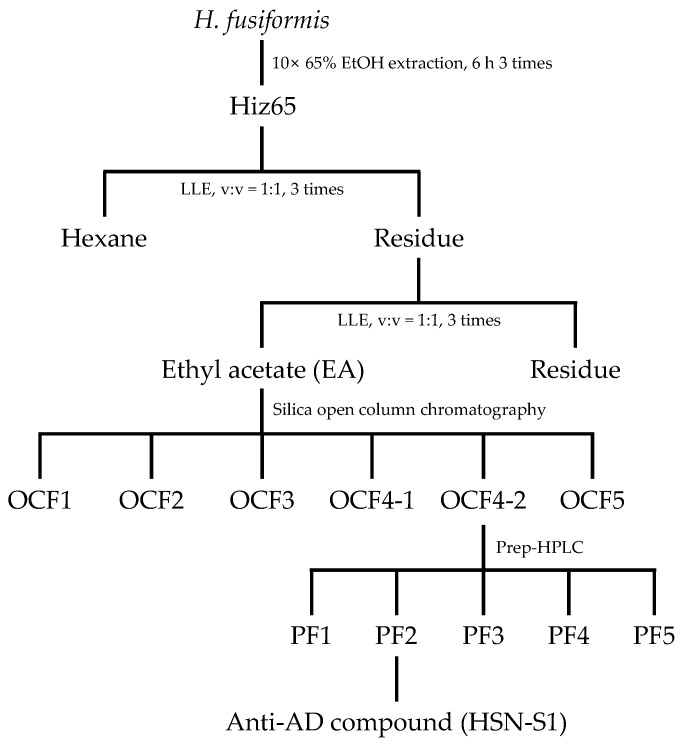
Optimized fractionation extraction process for isolating HSN-S1 with anti-AD properties.

The previously identified anti-AD fraction was preprocessed using silica gel column chromatography to separate the active compounds. Compounds were identified via staining with iodine fumes during the thin layer chromatography (TLC) and silica gel column processes (results shown in Figure S3), and absorbance was measured at 203 nm for HPLC and prep-HPLC. The compound containing anti-AD, OCF4-2, was ultimately purified using prep-HPLC (purification conditions are shown in Table S4). Both TLC and HPLC were employed to systematically assess the chemical profile of *H. fusiformis* extracts. This established a solid basis for the subsequent isolation and characterization of potential anti-AD compounds (final purification where anti-AD activity was confirmed is illustrated schematically in Figure 12). The chemical reagents for these processes were primarily sourced from Sigma-Aldrich (St. Louis, MO, USA), and analytical-grade solvents were obtained from DUKSAN (Seoul, Republic of Korea); the compounds were chosen for their purity and reliability to ensure the integrity of the chromatographic analyses.

#### 3.1.3. NMR and Mass Spectrometry Analysis

A Bruker Avance III HD 700 MHz instrument (Bruker BioSpin, Rheinstetten, Germany) was used to record NMR spectra. The system featured a 5 mm cryoprobe chilled with helium with z-axis gradients. Its automatic tuning and matching capabilities ensured precise and consistent spectrum data. The spectrometer was set to a resonance frequency of 700.40 MHz for proton (^1^H) NMR and 176.12 MHz for carbon (^13^C) NMR analyses to structurally elucidate the compounds of interest in detail. In addition to NMR spectroscopy, high-resolution mass spectrometry was performed to complement the structural insights obtained from NMR spectroscopy. The high-resolution mass spectrometry setup comprised an Ultimate 3000 UHPLC system (Thermo Fisher Scientific) integrated with an ESI-Qq-TOF mass spectrometer (micrOTOF-Q II, maXis HD, Bruker BioSpin), which provided unparalleled sensitivity and accuracy in mass determination. MS1 scans were executed over a mass-to-charge (m/z) range of 150–1500 to ensure a broad detection window for analyzing molecular ions. The most abundant ions underwent MS2 scans using high-energy collisional dissociation at 55.0 eV normalized collision energy.

### 3.2. In Vitro Assays for Immunomodulatory and Anti-Bacterial Activity

#### 3.2.1. Culture of AD-Related Cell Lines

HaCaT keratinocytes, RAW264.7, and Jurkat cells were utilized to study skin health and immune responses in AD. HaCaT cells received from the Korea Institute of Science and Technology (KIST, Gangneung, Republic of Korea), while RAW264.7 and Jurkat cells were from the Korea Cell Line Bank (KCLB, Seoul, Republic of Korea). HaCaT and Jurkat cells grew in Roswell Park Memorial Institute 1640 (RPMI1640) medium, and RAW264.7 in Dulbecco’s Modified Eagle’s Medium (DMEM). All media contained 10% fetal bovine serum (Gibco, Billings, MT, USA) and antibiotics (100 U/mL penicillin, 100 μg/mL streptomycin, Gibco). Cells were cultured at 37 °C with 5% CO_2_. FK506 (tacrolimus, Sigma) was used as a drug comparison group, serving as an immunosuppressant to inhibit immune overactivation.

#### 3.2.2. Splenocyte Culture

Splenocytes were isolated from BALB/c mouse spleens and processed in cold phosphate-buffered saline to obtain a single-cell suspension. Red blood cells were removed with a lysis buffer (eBioscience, San Diego, CA, USA) to preserve lymphocytes, according to the manufacturer’s protocol. Purified splenocytes were then cultured in RPMI 1640 medium enriched with 10% fetal bovine serum and antibiotics; this provided an environment for immune cell studies.

#### 3.2.3. THP-1 Lucia™ NF-κB and Jurkat Lucia™ NFAT Luciferase Assay

THP-1 Lucia™ NF-κB cells (InvivoGen, San Diego, CA, USA) were engineered to express luciferase under an NF-κB-responsive promoter. We pretreated cells with fractions, then stimulated them with LPS from *Escherichia coli* (Sigma-Aldrich, strain O55:B5) to activate NF-κB signaling. Luciferase activity, which indicated NF-κB activation, was quantified using the QUANTI-Luc assay solution (InvivoGen). Like the NF-κB assay, Jurkat Lucia™ NFAT cells (InvivoGen) were used to assess NFAT pathway activation. After HSN-S1 treatment, cells were stimulated with ConA (Sigma-Aldrich), and luciferase activity was measured, to evaluate NFAT signaling activation.

#### 3.2.4. NO and Antimicrobial Susceptibility Assay

The anti-inflammatory potential of HSN-S1 was evaluated in PGN-stimulated RAW264.7 cells via measuring NO production. We measured nitrite levels in the culture medium as an indicator of NO production using the Griess reaction (Sigma-Aldrich). The anti-bacterial activity against *S. aureus* was assessed to determine AD-related infections. *S. aureus* cultures were standardized using the McFarland scale to ensure consistent initial concentrations across all the experimental setups. We assessed HSN-S1’s minimal inhibitory concentrations (MICs) using the broth microdilution method. Post-treatment bacterial viability was quantified using a Microbial Viability Assay Kit (Dojindo, Tokyo, Japan).

#### 3.2.5. Quantitative Reverse Transcription Polymerase Chain Reaction (qRT-PCR) Analysis

Gene expression analyses were performed using qRT-PCR to determine the impact of HSN-S1 on specific immune responses and AD pathogenesis-related genes. Total RNA was isolated from the treated cell lines using TRIzol reagent (Thermo Fisher Scientific) according to the manufacturer’s guidelines. RNA purity and concentration were assessed using spectrophotometry. We reverse transcribed 1.5 µg of total RNA from each sample into cDNA using the ImProm-II Reverse Transcriptase System (Promega, Madison, WI, USA). This involved an incubation step at 42 °C for 1 h, followed by enzyme deactivation at 70 °C for 15 min to prepare the cDNA for amplification. The SensiMix™ SYBR Hi-ROX PCR Master Mix (Bioline, London, UK) was used on a Rotor-Gene 6000 real-time PCR system (Qiagen, Hilden, Germany) to perform qRT-PCR. The cycling conditions were as follows: initial denaturation at 95 °C for 15 min followed by 45 cycles of denaturation at 95 °C for 15 s (denaturation), annealing at 52 °C for 15 s (annealing), and extension at 72 °C for 10 s (extension). Specific primer pairs for the target genes were designed based on the gene sequences of interest, as listed in Table S4. We calculated relative target gene expression using the 2^−ΔΔCt^ method, with β-actin as the internal normalization control.

#### 3.2.6. Western Blot Analysis

For protein analysis, cell lysates were prepared using a 1% protein extraction solution containing protease and phosphatase inhibitors (Elpis Biotech, Daejeon, Republic of Korea; Roche, Basel, Switzerland). BCA Protein Assay Kits (Thermo Fisher Scientific) were used to determine protein concentrations. Proteins (50 µg per sample) were separated with 10% sodium dodecyl sulfate-polyacrylamide gel electrophoresis and transferred to polyvinylidene difluoride membranes (Cytiva, Basel, Switzerland). Membranes were blocked with 5% skim milk (BD Biosciences, Franklin Lakes, NJ, USA) before incubation with primary antibodies targeting phosphorylated extracellular signal-regulated kinase (p-ERK) and phosphorylated p38 (p-p38). Horseradish peroxidase-conjugated secondary antibodies were used for protein detection. Protein bands were detected using SuperSignal West Dura Extended Duration Substrate (Thermo Fisher Scientific) and captured on an Agfa X-ray film (Agfa, Mortsel, Belgium).

#### 3.2.7. Confocal Microscopy Analysis

To prepare and stain cells, RAW264.7 macrophages and Jurkat T cells were treated with 4% paraformaldehyde for 15 min at room temperature to maintain their cellular architecture. After fixation, the cells were blocked with 5% bovine serum albumin for 2 h to reduce non-specific binding and enhance antibody penetration. The cells were incubated with primary antibodies against NF-κB p50 and NFATc2, key transcription factors in immune regulation. Following primary antibody incubation, the cells were exposed to species-matched secondary antibodies conjugated with tetramethylrhodamine for 1 h to allow fluorescence detection. Nuclei were counterstained with NucBlue™ Live Cell Stain ReadyProbes™ (Invitrogen Corporation, Carlsbad, CA, USA) to distinguish cell nuclei. The stained cells were then mounted on slides using a Micromount microscope (Leica, Wetzlar, Germany) to preserve fluorescence, and were imaged using a Stellaris 5 confocal microscope (Leica). Images were analyzed with Las X Office 1.4.5 software (Leica).

### 3.3. In Vivo Efficacy Evaluation

#### 3.3.1. Animal Care and AD-like Skin Lesion Induction

**Figure 13:**
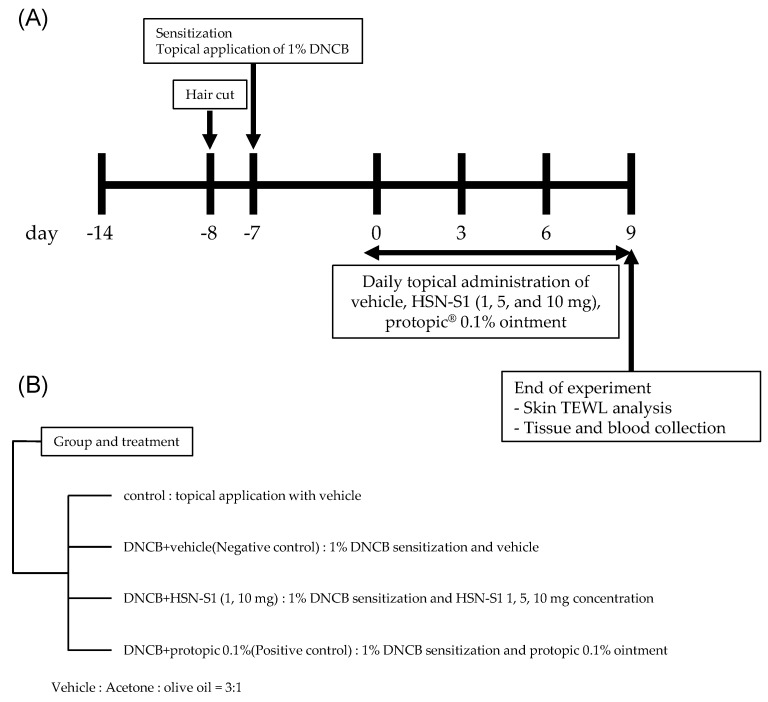
Overview of the HSN-S1 efficacy study protocol in a DNCB-induced AD mouse model. (**A**) The schematic timeline outlines the key stages from the initial induction of AD-like lesions with DNCB to the maintenance of these conditions for a consistent evaluation period. (**B**) The treatment groups were categorized, detailing the HSN-S1 administration at doses of 1, 5, and 10 mg, with a control group receiving 0.1% Protopic ointment, a benchmark AD treatment.

The Gangneung National University Institutional Animal Care and Use Committee granted approval for this study (No. GWNU2022-4), which complied with all applicable ethical guidelines for animal research. Experimental animal creation and procedures are schematically represented in Figure 13. Seven-week-old pathogen-free BALB/c male mice, obtained from KOATECH (Gyeonggi, Republic of Korea), were housed under controlled conditions with a consistent temperature of 23 ± 2 °C, relative humidity of 50 ± 20%, a 12/12-h light/dark cycle, and four daily air changes. The mice had access to RodFeed (DBL, Chungbuk, Republic of Korea) and 0.5 μm filtered water ad libitum throughout the study duration. To model AD-like lesions, the mice were divided into six groups based on the treatment received: an untreated control group (ctrl), a 2,4-dinitrochlorobenzene (DNCB)-induced AD group treated with vehicle only (negative control, NC), a DNCB-induced AD group treated with 0.1% Protopic^®^ ointment (positive control, PC), and three DNCB-induced AD groups treated with varying HSN-S1 doses (1, 5, and 10 mg). The negative control group received a vehicle solution applied topically to the dorsal skin for 13 days to simulate AD, without therapeutic intervention. The positive control group received a topical application of 0.1% Protopic^®^ ointment as the standard AD treatment. The experimental groups were topically treated with HSN-S1 at specified doses for 13 days to evaluate their anti-AD effects. AD-like skin lesions were induced using DNCB, which was applied ectopically to the dorsal skin of mice. Initially, 200 µL of 1% DNCB in acetone/corn oil (3:1) was applied for 1 week to sensitize the skin, followed by a maintenance phase involving weekly applications of 200 µL of 0.5% DNCB.

#### 3.3.2. Histological Analysis

Skin tissues were preserved in 10% neutral buffered formalin for 72 h to ensure proper fixation. After fixation, tissues underwent dehydration, clearing, and paraffin embedding. A microtome was used to cut 5 μm thick sections from the embedded tissues. Hematoxylin and eosin staining revealed cellular and structural details of the skin. Hematoxylin and eosin staining highlighted the epidermal and dermal layers, which facilitated examining histopathological changes such as epidermal thickening, cellular infiltration, and integrity of the skin barrier. Stained sections were examined under an optical microscope at 200× magnification. Epidermal thickness was measured in three random areas per section to quantitatively assess the treatment effects on skin structure. ImageJ (National Institutes of Health, Bethesda, MD, USA) software was used to perform measurements.

#### 3.3.3. TEWL Analysis

TEWL was measured using a Gpskin barrier (GPskin, Seoul, Republic of Korea), a device designed to assess the volume of water that evaporates from the skin surface. Measurements were taken at the center of the lesioned area to ensure consistency and accuracy. The device was carefully positioned against the skin to avoid pressure that could alter natural TEWL values. Readings were taken at least twice for each mouse to minimize variability and ensure data reliability. TEWL values were recorded in grams per hour per square meter (g/h/m²); this provided a quantitative measure of the skin’s ability to retain moisture and function as an effective barrier.

#### 3.3.4. Serum IgE Measurement in Blood

Following the treatment period and induction of AD-like lesions, the mice were euthanized, and blood samples were collected in serum separator tubes. The blood was allowed to clot at room temperature for 30 min before centrifugation at 1500× *g* for 20 min. This process separated the serum from the clotted blood. The total serum IgE concentration was determined using a sandwich enzyme-linked immunosorbent assay (ELISA) kit designed for mouse IgE (Chondrex, Woodinville, WA, USA). The assay was conducted according to the manufacturer’s guidelines. ELISA plates were coated with a capture antibody, serum samples were added, and bound IgE was identified using a biotinylated detection antibody, followed by the addition of streptavidin-horseradish peroxidase. A substrate solution initiated the reaction, and optical density was measured at 450 nm using a microplate reader (Molecular Devices, San Jose, CA, USA). An ELISA kit was used to quantify IgE concentrations, utilizing a standard curve generated from known mouse IgE concentrations. The results were expressed in units of concentration (μg/mL); therefore, serum IgE levels across the different treatment groups could be compared.

### 3.4. Statistical Analysis

The data are reported as the mean ± standard error of the mean (SEM). Differences between experimental groups were analyzed for statistical significance using Student’s *t*-test or one-way analysis of variance (ANOVA), followed by Tukey’s post hoc test, as implemented in GraphPad Prism Version 5.01 (GraphPad Software Inc., La Jolla, CA, USA). Statistical significance was considered at *p* < 0.05.

## 4. Conclusion

Our investigation of HSN-S1, derived from *H. fusiformis*, reveals its significant potential as a novel therapeutic agent for AD. Through bioactivity-guided isolation, we identified HSN-S1 and extensively characterized its anti-inflammatory, immunomodulatory, and antibacterial properties.

Key findings demonstrate HSN-S1’s ability to (1) suppress pro-inflammatory cytokines, (2) modulate critical signaling pathways (ERK, p38 MAPK, NF-κB), (3) act on various AD-relevant cell types (keratinocytes, macrophages, CD4+ T cells), (4) inhibit *S. aureus* growth, a pivotal aspect of AD pathology, and (5) modulate cytokines through NFAT and NF-κB pathways. In vivo assessments further substantiated HSN-S1’s efficacy, revealing significant reductions in AD markers, including (1) epidermal thickening, (2) transepidermal water loss (TEWL), (3) serum IgE levels, and (4) Th2 and Th17 cytokines. These results affirm HSN-S1’s symptom-mitigating capabilities and efficacy against underlying immune dysregulation in AD.

Considering these compelling results, HSN-S1 represents a promising frontier in AD therapy, offering a comprehensive approach to addressing both immunological and microbial facets of the disease. The convergence of anti-inflammatory, immunomodulatory, and antibacterial actions in a single compound may revolutionize the treatment land-scape for AD patients. This foundational research not only paves the way for future clinical trials to explore HSN-S1’s therapeutic potential in humans but also provides novel insights and potential synergistic approaches to AD treatment. By situating HSN-S1 within existing immunomodulatory research, our study enhances the scientific dialogue on innovative therapies, offering hope for more effective and holistic AD management strategies in this challenging condition.

## Figures and Tables

**Table 1:** NMR spectral data for HSN-S1 in DMSO-*d*_6_.

Atom Position	^1^H NMR δH (ppm), Multiplicity (J in Hz)	^13^C NMR δC (ppm)
1	-	173.0
2	2.30, t (7.0)	33.4
3	1.54, quint (7.0)	26.4
4	1.34, quint (7.0)	28.5
5	2.05, t (7.0)	24.1
6	5.35–5.34, m	126.9
7	5.34–5.30, m	127.7
8	2.82–2.79, m	25.2
9	5.34–5.30, m	127.7
10	5.34–5.30, m	127.8
11	2.82–2.79, m	25.2
12	5.34–5.30, m	128.0
13	5.34–5.30, m	128.1
14	2.82–2.79, m	25.1
15	5.30–5.28, m	129.6
16	5.37–5.35, m	131.5
17	2.03, t (7.0)	20.0
18	0.92, t (7.7)	14.1
1’	4.28, dd (11.2, 2.8); 3.94, dd (11.2, 7.0)	66.9
2’	3.66, m	68.3
3’	3.55, d (9.1)	69.5
4’	3.55, d (9.1)	71.2
5’	3.45, m	71.2
6’	3.39, dd (11.2, 6.3); 3.61, dd (11.2, 3.5)	63.8

Note: Mult., multiplicity; d, doublet; t, triplet; dd, doublet of doublet; m, multiplet; quint, quintet. Chemical shifts (δ) are in parts per million (ppm), and coupling constants (J) are in hertz (Hz).

## Data Availability

Data are contained within the article.

## Supplementary Materials

The following supporting information can be downloaded at: https://www.mdpi.com/article/10.3390/md22080354/s1, Figure S1: Schematic representation of the extraction of biologically active fractions; Figure S2: Separation of fractions from *H. fusiformis* EA fraction using Prep-HPLC; Figure S3: TLC analysis results for Hizikia EA fraction using silica gel open column fractions; Figure S4: ^1^H NMR of HSN-S1 in DMSO-*d*6; Figure S5: ^13^C NMR of HSN-S1 in DMSO-*d*6; Figure S6: DEPT-90 of HSN-S1 in DMSO-*d*6; Figure S7: DEPT-135 of HSN-S1 in DMSO-*d*6; Figure S8: HSQC of HSN-S1 in DMSO-*d*6; Figure S9: COSY of HSN-S1 in DMSO-*d*6; Figure S10: HMBC of HSN-S1 in DMSO-*d*6; Figure S11: ^1^H NMR of mannitol standard in D_2_O; Figure S12: ^1^H NMR of the HSN-S1 sugar alcohol in D_2_O; Table S1: Results of cytotoxicity analysis on HSN-S1; Table S2: HPLC gradient elution conditions for *H. fusiformis* EA fraction; Table S3: Effect of different subfractions separated from *H. fusiformis* on the production of IL-2 in anti-CD3ε-induced splenocytes and expression of NF-κB in LPS-induced THP-1 Lucia™ NF-κB cells; Table S4: Elution conditions for silica gel open column chromatography (A) and Prep-HPLC gradient elution (B) for purification of anti-AD compounds; Table S5: Primer sequences for quantitative reverse transcription polymerase chain reaction.
