# Supplementary material for: Antibacterial and Immunosuppressive Effects of a Novel Marine Brown Alga-Derived Ester in Atopic Dermatitis
Source: Mar Drugs. 2024 Jul 30;22(8):354. doi: 10.3390/md22080354 (PMC11355872; doi:10.3390/md22080354)
Supplement: Supplementary file 1 [file marinedrugs-22-00354-s001.zip › marinedrugs-3127710-supplementary.pdf]

Supplementary Material

Antibacterial and Immunosuppressive Effects of a Novel Marine Brown Algae-Derived Ester in Atopic Dermatitis

Contents of Figures

Figure S1: Schematic representation of the extraction of biologically active fractions 4

Figure S2: Separation of fractions from *H. fusiformis* EA fraction using Prep-HPLC 4

Figure S3: TLC analysis results of *Hizikia* EA fraction using silica gel chromatography fractions 5

Figure S4: ^{1}H NMR of HSN-S1 in DMSO-d_6 6

Figure S5: ^{13}C NMR of HSN-S1 in DMSO-d_6 6

Figure S6: DEPT-90 of HSN-S1 in DMSO-d_6 7

Figure S7: DEPT-135 of HSN-S1 in DMSO-d_6 7

Figure S8: HSQC of HSN-S1 in DMSO-d_6 8

Figure S9: COSY of HSN-S1 in DMSO-d_6 8

Figure S10: HMBC of HSN-S1 in DMSO-d_6 9

Figure S11: ^{1}H NMR of mannitol standard in D_2O 9

Figure S12: ^{1}H NMR of the HSN-S1 sugar alcohol in D_2O 10

Contents of Tables

Table S1: Results of cytotoxicity analysis on HSN-S1 11

Table S2: HPLC gradient elution conditions for *H. fusiformis* EA fraction 11

Table S3: Effect of different subfractions separated from *H. fusiformis* on the production of IL-2 in anti-CD3ε-induced splenocytes and expression of NF-κB in LPS-induced THP-1 Lucia™ NF-κB cells 11

Table S4: Elution conditions for silica gel open column chromatography (A) and Prep-HPLC gradient elution (B) for purification of anti-AD compounds 12

Table S5: Primer sequences for quantitative reverse transcription polymerase chain reaction 13

Supplementary data

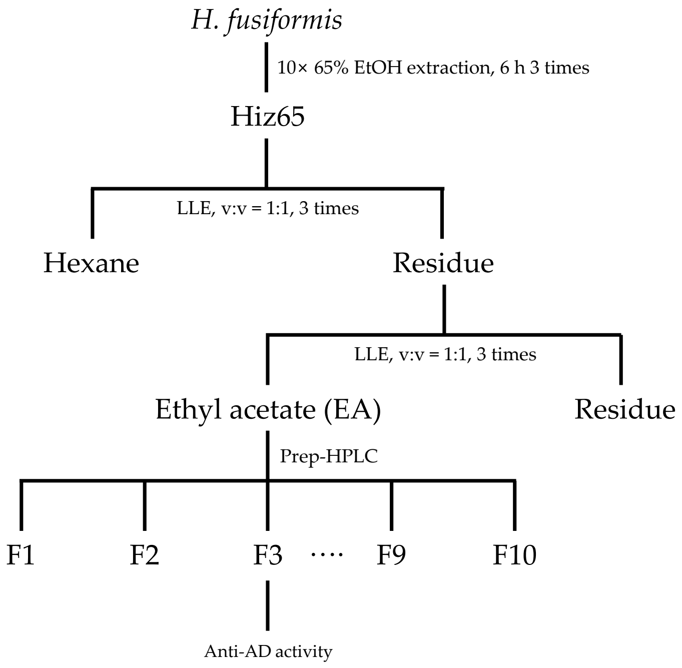

Figure S1: Schematic representation of the extraction of biologically active fractions.

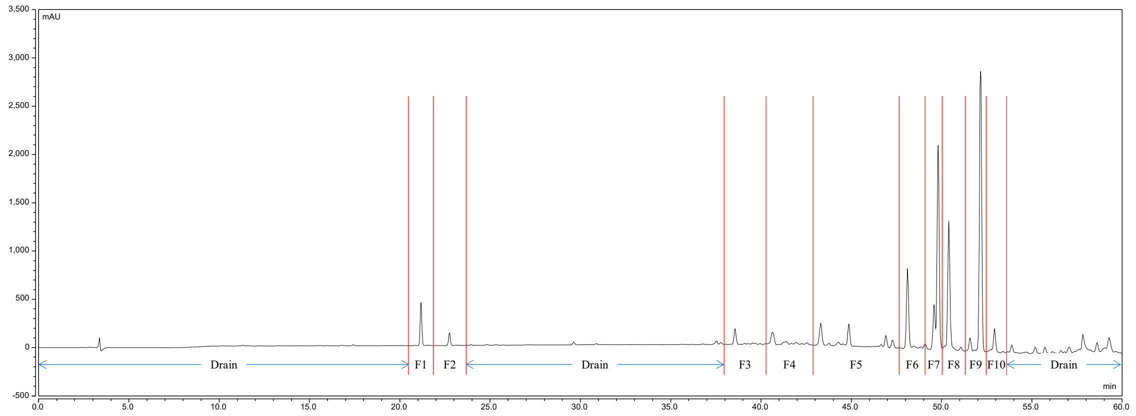

Figure S2: Separation of fractions from *H. fusiformis* EA fraction using Prep-HPLC. "Drain section" refers to areas where no peak is detected during the washing phase.

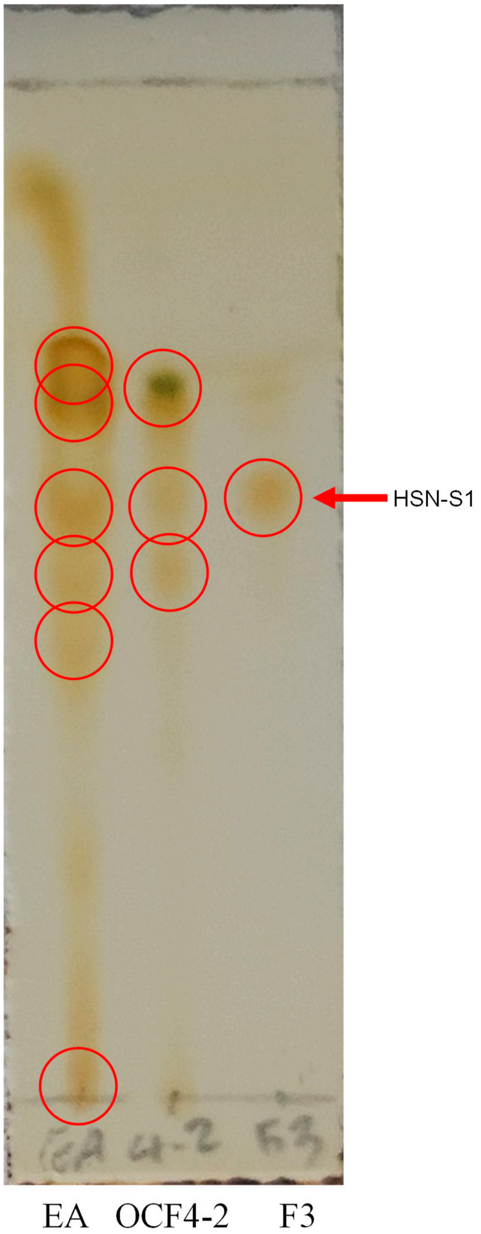

Figure S3: TLC analysis results of *Hizikia* EA fraction using silica gel chromatography fractions.

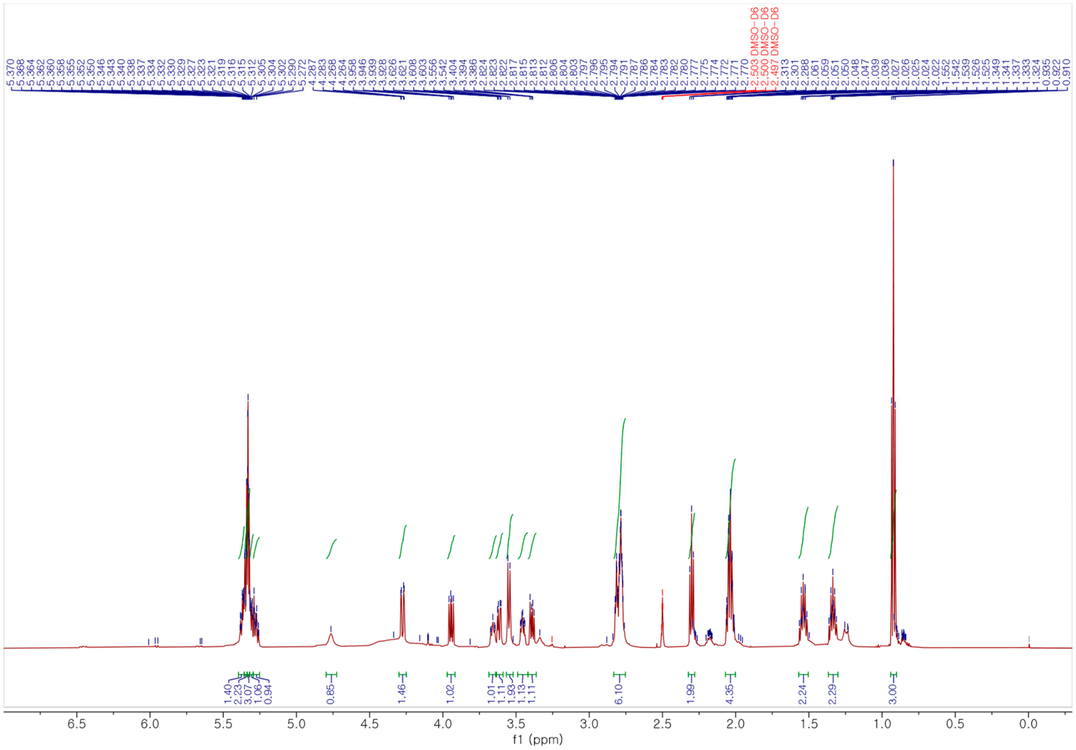

Figure S4: ^{1}H NMR of HSN-S1 in DMSO-d_6.

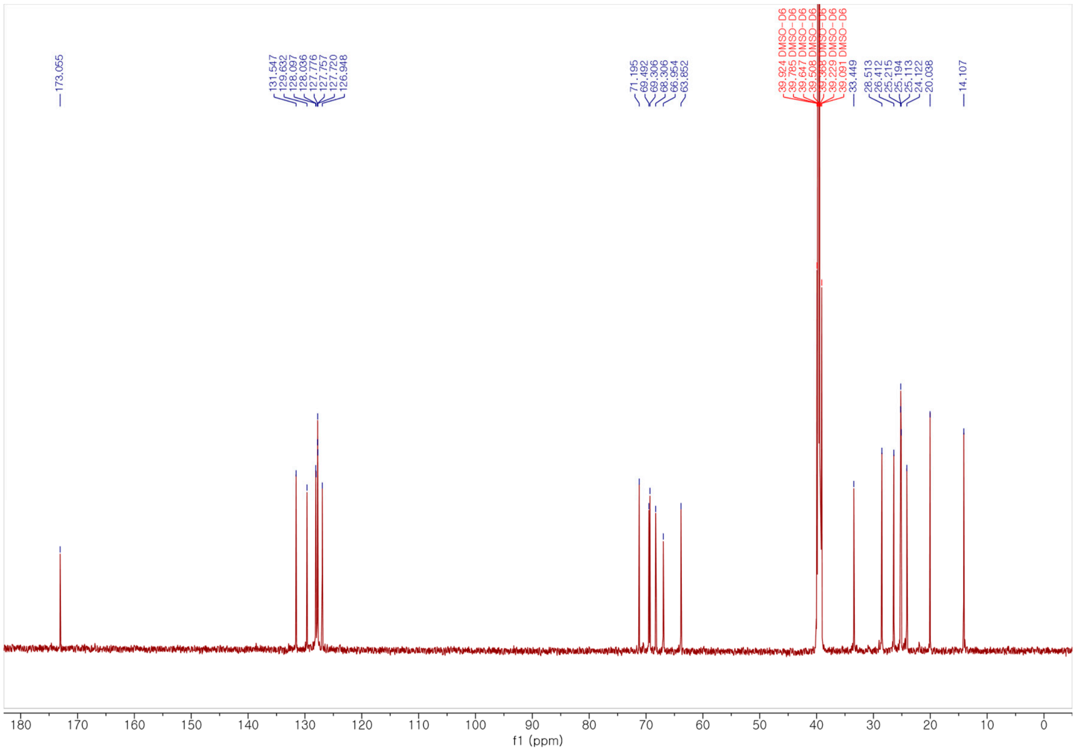

Figure S5: ^{13}C NMR of HSN-S1 in DMSO-d_6.

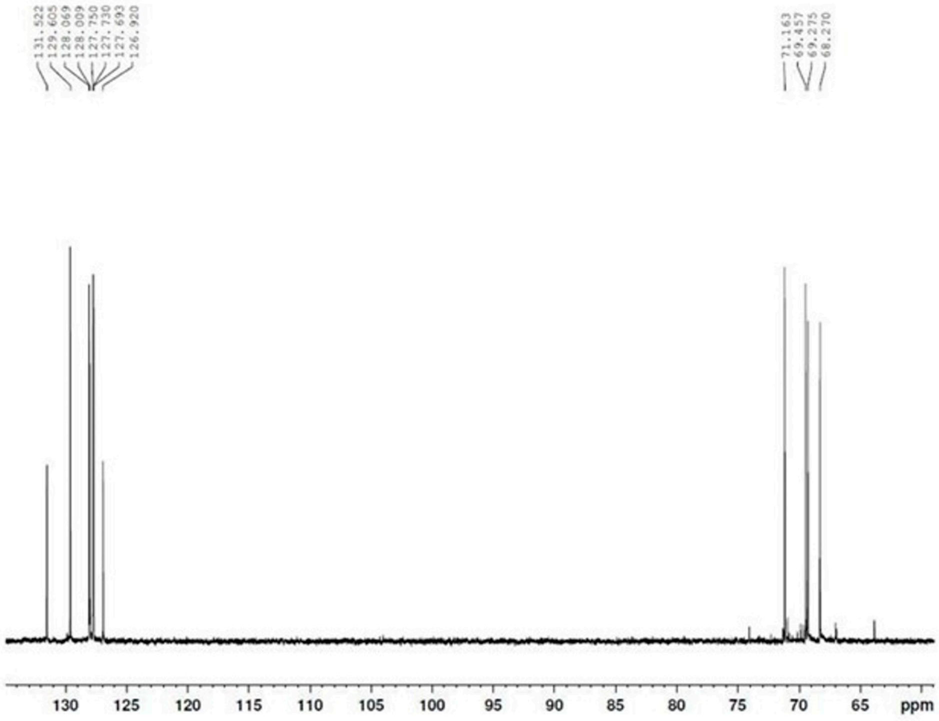

Figure S6: DEPT-90 of HSN-S1 in DMSO-d_6.

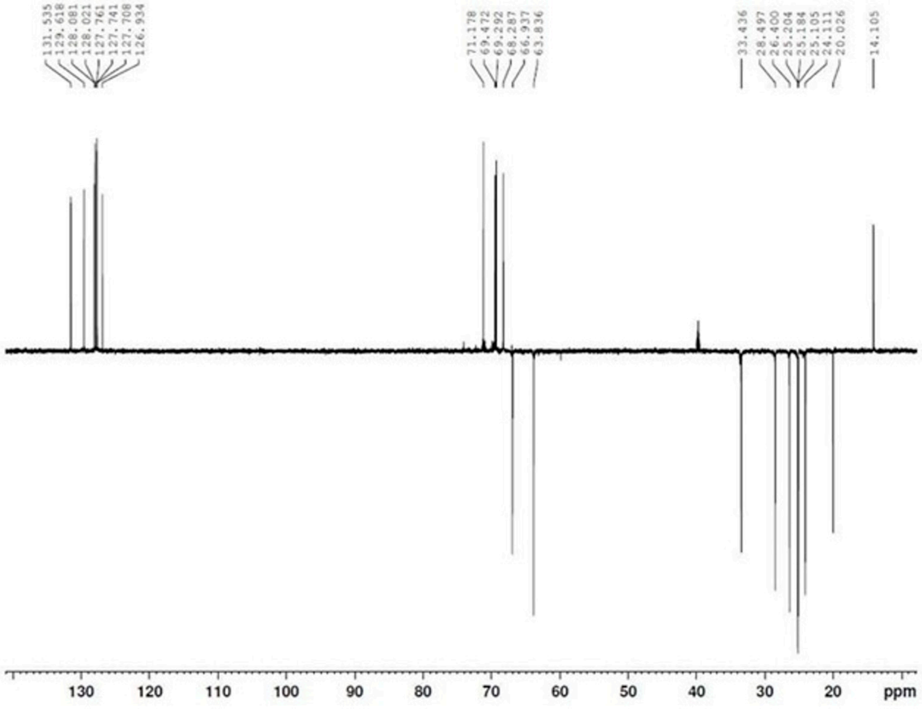

Figure S7: DEPT-135 of HSN-S1 in DMSO-d_6.

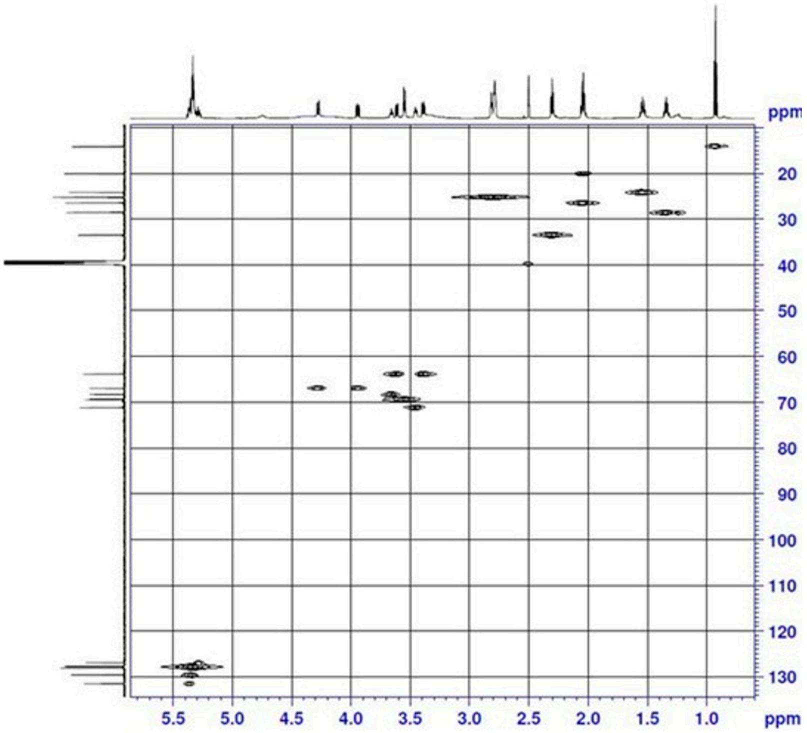

Figure S8: HSQC of HSN-S1 in DMSO-*d6*.

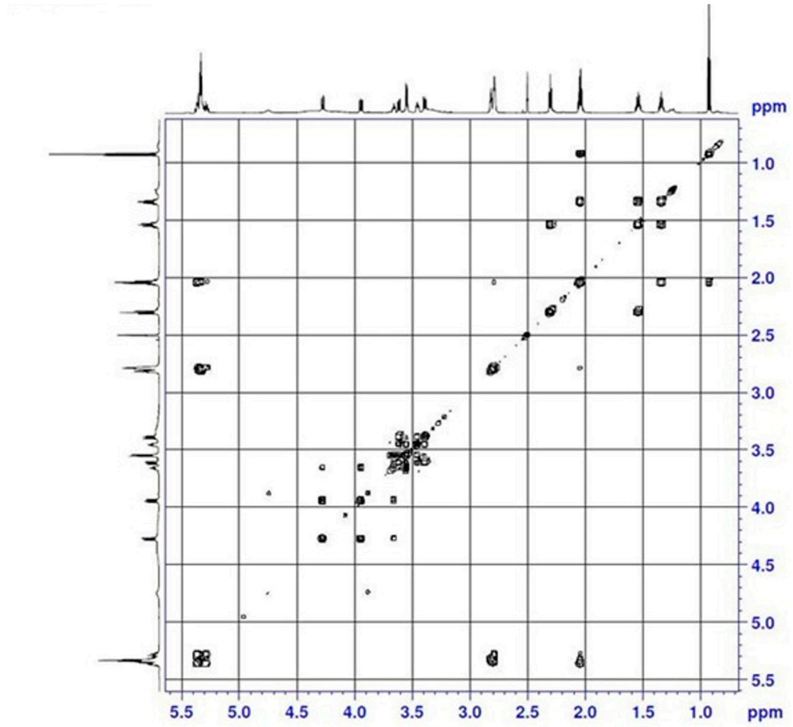

Figure S9: COSY of HSN-S1 in DMSO-*d6*.

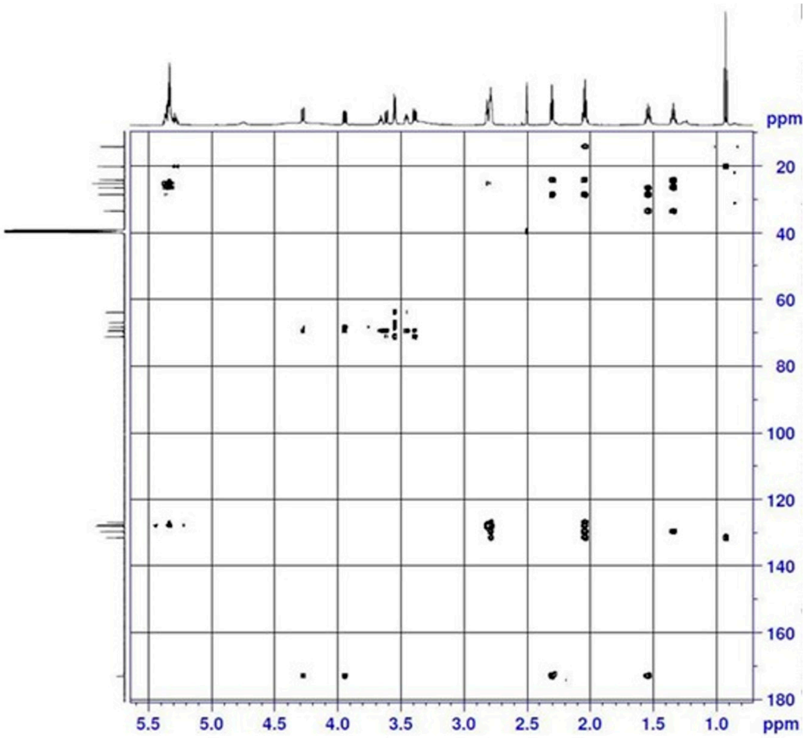

Figure S10: HMBC of HSN-S1 in DMSO-d_6.

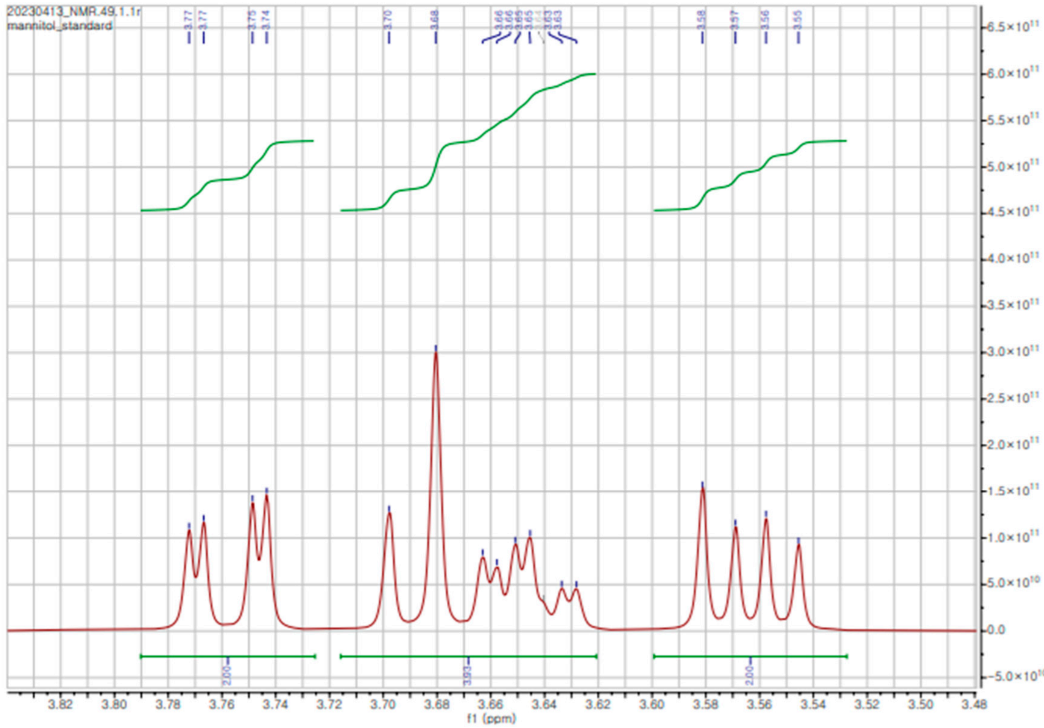

Figure S11: ^{1}H NMR of mannitol standard in D_2O.

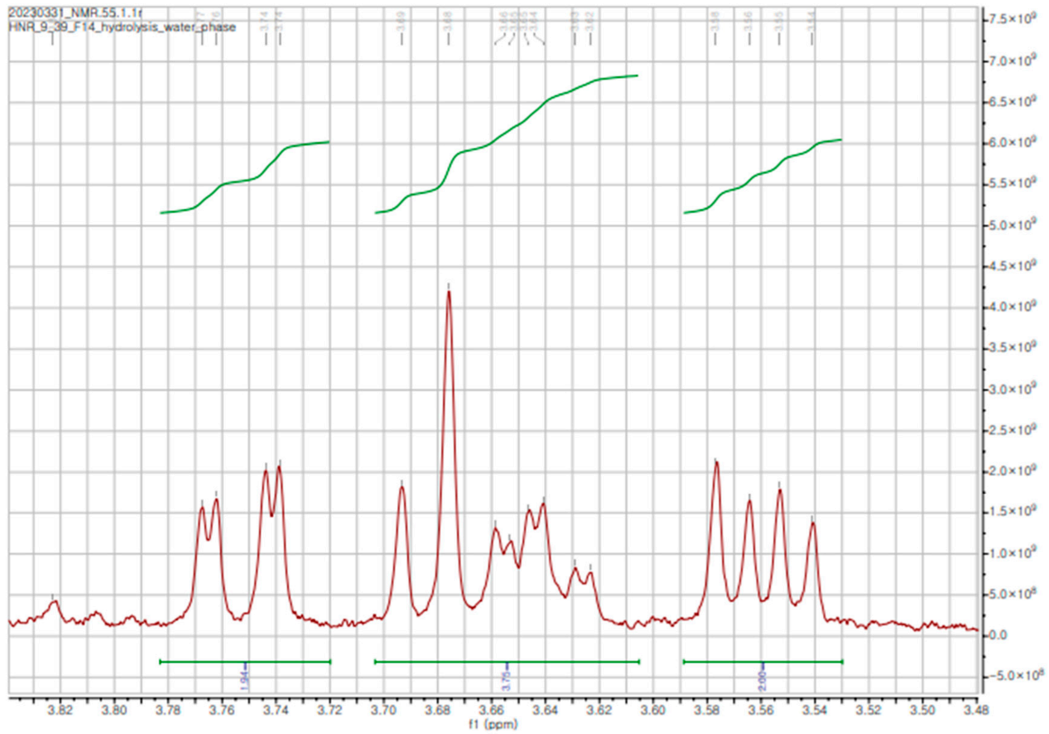

Figure S12: ^{1}H NMR of the HSN-S1 sugar alcohol in D_2O.

Table S1: Results of cytotoxicity analysis on HSN-S1.

Cell	Dose range for cell treatment (μg/mL)	CC_{50} (μg/mL)	Remarks
HaCaT	1.0 to 100.0	24.2 ± 0.1	
RAW264.7	1.0 to 100.0	54.4 ± 1.8	
Splenocytes	1.0 to 100.0	62.8 ± 0.2	
Naïve $CD4^+$ Th	1.0 to 100.0	> 100	
Jurkat	1.0 to 100.0	50.3 ± 0.1	
RBL-2H3	0.3 to 100	14.6 ± 0.4	
Jurkat Lucia-NFAT	1.0 to 100.0	57.9 ± 2.8	Luciferase activity of transfected cells

Table S2: HPLC gradient elution conditions for *H. fusiformis* EA fraction.

Time (min)	Flow (mL/min)	% D.W.	% ACN
0.0	1.0	100.0	0.0
53.0	1.0	0.0	100.0
58.0	1.0	0.0	100.0
60.0	1.0	100.0	0.0

D.W: distilled water, ACN: acetonitrile.

Table S3: Effect of different subfractions separated from *H. fusiformis* on the production of IL-2 in anti-CD3ε-induced splenocytes and expression of NF-κB in LPS-induced THP-1 Lucia™ NF-κB cells.

Fraction No	Splenocytes (% of Anti-CD3ε group)	THP-1 NFκB (% of LPS group)
F1	48.13 ± 0.01	36.03 ± 0.63
F2	39.05 ± 0.66	71.34 ± 0.19
**F3**	**30.53 ± 0.15**	**20.48 ± 0.38**
F4	39.34 ± 2.04	80.77 ± 0.48
F5	65.69 ± 4.09	31.24 ± 0.10
F6	45.24 ± 3.76	84.10 ± 0.30
F7	54.63 ± 6.29	84.07 ± 0.32
F8	75.06 ± 8.15	76.69 ± 0.94
F9	106.55 ± 4.11	61.46 ± 0.57
F10	61.22 ± 2.63	80.26 ± 0.87

Table S4: Elution conditions for silica gel open column chromatography (A) and Prep-HPLC gradient elution (B) for purification of anti-AD compounds.

(A)

Fraction No	Solvent	BV
OCF1	DCM	2.5
OCF2	DCM:EA=1:1	2.5
OCF3	EA	2.5
OCF4-1	DCM:MeOH=7:1	2.5
OCF4-2	DCM:MeOH=7:1	2.5
OCF5 (wash)	MeOH	2.5

BV: bed volume, DCM: dichloromethane, EA: ethyl acetate, and MeOH: methanol.

(B)

Time (min)	Flow (mL/min)	% D.W	% ACN
0.0	1.0	50.0	50.0
53.0	1.0	0.0	100.0
58.0	1.0	0.0	100.0
60.0	1.0	50.0	50.0

D.W: distilled water and ACN: acetonitrile.

Table S5: Primer sequences for quantitative reverse transcription polymerase chain reaction.

Species	Gene	Direction	Sequence (5′ to 3′)	Accession
Human	TNF-α	Forward	5′- CTC TTC TCC TTC CTG ATC GT	NM_000594.4
		Reverse	5′- CTG GTT ATC TCT CAG CTC CA	
	MCP-1	Forward	5′- TAG AAG AAT CAC CAG CAG CA	NM_002982.4
		Reverse	5′- GTC TTC GGA GTT TGG GTT TG	
	CXCL10	Forward	5′- CTA AGT GGC ATT CAA GGA GT	NM_001565.4
		Reverse	5′- AGA CCT TTC CTT GCT AAC TG	
	β-actin	Forward	5′- ACC TGA CTG ACT ACC TCA TG	NM_001101.5
		Reverse	5′- CTC ATT GCC AAT GGT GAT GA	
Mouse	IL-1β	Forward	5′- CTG TGT AAT GAA AGA CGG CA	NM_008361.4
		Reverse	5′- TAT GTC CTG ACC ACT GTT GT	
	IL-2	Forward	5′- CAC TTC AAG CTC CAC TTC AA	NM_008366.3
		Reverse	5′- AGT CAA ATC CAG AAC ATG CC	
	IL-4	Forward	5′- TGA GAG AGA TCA TCG GCA TT	NM_021283.2
		Reverse	5′- TGA TGT GGA CTT GGA CTC AT	
	IL-9	Forward	5′- TGA CAT ACA TCC TTG CCT CT	NM_008373.2
		Reverse	5′- AGT CTT GAT TTC TGT GTG GC	
	IL-10	Forward	5′- ACA ATA ACT GCA CCC ACT TC	NM_010548.2
		Reverse	5′- CCA CTG CCT TGC TCT TAT TT	
	IL-13	Forward	5′- TCT GAC CCT TAA GGA GCT TA	NM_008355.3
		Reverse	5′- TAC AGA GGC CAT GCA ATA TC	
	IL-17A	Forward	5′- CTC CAG AAT GTG AAG GTC AA	NM_010552.3
		Reverse	5′- AAC AGA ATT CAT GTG GTG GT	
	IL-21	Forward	5′- CCT GAA CTT CTA TCA GCT CC	NM_021782.3
		Reverse	5′- ATC ACA GGA AGG GCA TTT AG	
	IL-22	Forward	5′- CTC CCC CAG TCA GAC AGG TT	NM_016971.2
		Reverse	5′- AAA CAG CAG GTC CAG TTC CC	
	IL-33	Forward	5′- CTG TTG ACA CAT TGA GCA TC	NM_001360725.1
		Reverse	5′- CCA GAT GTC TGT GTC TTT GA	
	COX-2	Forward	5′- CGA TGT CAT GGA ACT GTA CC	NM_011198.5
		Reverse	5′- ATC TTA AAA CCC ACT TCG CC	
	GATA3	Forward	5′- TCG TAC ATG GAA GCT CAG TA	XM_030247533.2
		Reverse	5′- AAG AGA TGA GGA CTG GAG TG	
	β-actin	Forward	5′-CAT CAA AGA GAA GCT GTG CT	NM_007393.5
		Reverse	5′-GAA GGA AGG CTG GAA AAG AG	
